# Comparative evaluation of video-based on-line course versus serious game for training medical students in cardiopulmonary resuscitation: A randomised trial

**DOI:** 10.1371/journal.pone.0214722

**Published:** 2019-04-08

**Authors:** David P. de Sena, Daniela D. Fabrício, Vinícius D. da Silva, Luiz Carlos Bodanese, Alexandre R. Franco

**Affiliations:** 1 School of Health Sciences, Post-Graduate Program in Health Sciences, Pontifícia Universidade Católica do Rio Grande do Sul (PUCRS), Porto Alegre, Rio Grande do Sul, Brazil; 2 Department of Otolaryngology, PUCRS, Porto Alegre, Rio Grande do Sul, Brazil; 3 Department of Pathological Anatomy, Hospital de Câncer de Barretos, Barretos, São Paulo, Brazil; 4 Department of Cardiology, PUCRS, Porto Alegre, Rio Grande do Sul, Brazil; 5 Brain Institute of Rio Grande do Sul, PUCRS, Porto Alegre, Rio Grande do Sul, Brazil; University of Palermo, ITALY

## Abstract

**Purpose:**

To estimate the effect size of a serious game for cardiopulmonary resuscitation (CPR) training in comparison with a video-based on-line course in terms of learning outcomes among medical students before simulation-based CPR using a manikin.

**Methods:**

Participants were 45 first-year medical students randomly assigned to CPR self-training using either a video-based Apple Keynote presentation (*n* = 22) or a serious game developed in a 3D learning environment (*n* = 23) for up to 20 min. Each participant was evaluated on a written, multiple-choice test (theoretical test) and then on a scenario of cardiac arrest (practical test) before and after exposure to the self-learning methods. The primary endpoint was change in theoretical and practical baseline scores during simulated CPR. This study was conducted in 2017.

**Results:**

Both groups improved scores after exposure. The video group had superior performance in both the theoretical test (7.56±0.21 vs 6.51±0.21 for the game group; *p* = 0.001) and the practical test (9.67±0.21 vs 8.40±0.21 for the game group; *p* < 0.001). However, students showed a preference for using games, as suggested by the longer time they remained interested in the method (18.57±0.66 min for the game group vs 7.41±0.43 for the video group; *p* < 0.001).

**Conclusions:**

The self-training modality using a serious game, after a short period of exposure, resulted in inferior students’ performance in both theoretical and practical CPR tests compared to the video-based self-training modality. However, students showed a clear preference for using games rather than videos as a form of self-training.

## Introduction

Simulation training is considered essential for learning cardiopulmonary resuscitation (CPR) [1, 2], a life-saving technique in the presence of cardiac arrest, a leading cause of death in many countries [3–5]. Since 2015, the American Heart Association (AHA) has recommended the use of high-fidelity manikins, simulators, feedback devices, and on-line training courses as resources for teaching and learning CPR [2, 6, 7]. Studies have shown that practice on manikins under instructor supervision is the most effective training modality [8, 9]. However, the implementation of face-to-face instructor-led training programs using manikins remains limited by the lack of resources [10, 11]. In addition, the quality of CPR manoeuvres by students trained using a traditional instructor-led CPR course as compared to students trained with self-directed CPR program, without instructor involvement, has been reported as similar [12–14].

Previous studies show that the use of video-based training is effective in teaching medical content [15–17]. Most video-based CPR courses use a short PowerPoint presentation with step-by-step voice-over narration, which can be easily shared over a network. These on-line CPR courses have been successful in enhancing learning, as well knowledge, skills, and behaviours related to the management of cardiac arrest [18]. Satisfactory results have also been obtained with CPR training based on serious games, i.e., games designed for a specific purpose beyond entertainment. [19–23].

Both video-based courses and serious games have been used to pretrain medical students before simulation training, but only one study has compared the effectiveness of these two modalities [24]. However, it remains unclear which of the two options is more efficient and better accepted by students in the process of knowledge acquisition. Therefore, the current pilot trial was set up to estimate the effect size of a serious game for CPR training in comparison with a video-based on-line course in terms of learning outcomes among first-year medical students. We tested the hypothesis that students trained with a serious game would perform better in a CPR simulation session than those trained with a video-based on-line course.

## Methods

In order to compare the learning outcomes of a video-based on-line course with a simulation-based CPR training game, a prospective randomised controlled trial was conducted in the Department of Medical Skills at the Pontifícia Universidade Católica do Rio Grande do Sul (PUCRS) School of Medicine in Porto Alegre, southern Brazil. The study was approved by the Institutional Review Board of PUCRS (IRB number 1818537, July 28, 2014) (S1 Document). All procedures were in accordance with the 1964 Helsinki declaration and its later amendments or comparable ethical standards. The study followed the CONSORT guidelines for the reporting of simulation-based randomised controlled trials (S1 Checklist). Following institutional guidance, this clinical trial was retrospectively registered at ClinicalTrials.gov with identifier NCT03729037 (https://clinicaltrials.gov/ct2/show/NCT03729037). The authors confirm that all ongoing and related trials for this intervention are registered.

### Participants and randomisation

Participants were first-year medical students from PUCRS School of Medicine who voluntarily participated in the study. Recruitment initiated on August 10, 2016, and the trial was completed by June 20, 2017. Eligible participants were all students aged 18 years or over who had never participated in CPR training or whose last training was more than 5 years prior to the study. Students were invited to participate via e-mail or directly while attending their regular courses. Informed consent was obtained from all individual participants included in the study. Demographic data were collected from all participants at study entry.

For allocation of the participants, a computer-generated random number list (http://www.randomization.com) was prepared by an investigator with no involvement in the trial. Participants were randomly assigned, with a 1:1 allocation ratio, to one of two groups for CPR training before a simulation session on CPR: the ‘video’ group and the ‘game’ group.

### Study design and interventions

**Fig 1:**
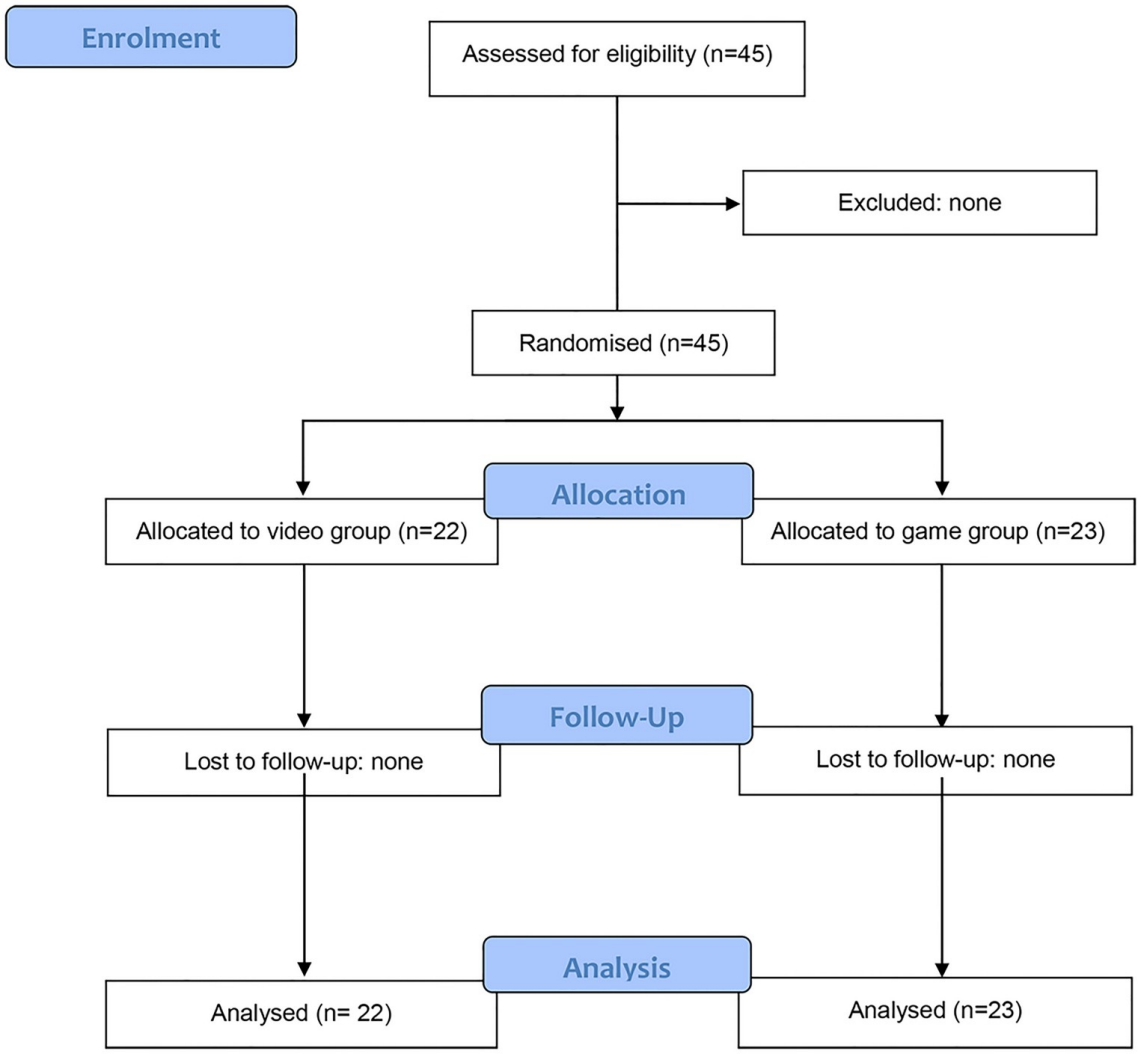
Flow diagram of participant selection.

The process of participant selection is shown in Fig 1. Before the start of the trial, in order to assess the participants’ baseline level, they were evaluated individually for their theoretical knowledge on a written, 10-question, multiple-choice test (theoretical pre-test) and for their practical performance on a 10-min simulated scenario of cardiac arrest using a CPR training manikin (practical pre-test).

Then, immediately following the baseline assessment, the participants randomised to the video group watched a video-recorded lecture on the management of adult cardiac arrest, while the participants randomised to the game group played a serious game on the same topic. Both were developed based on the 2015 AHA Guidelines for CPR and Emergency Cardiovascular Care (ECC) [2]. All participants received an Apple iPad 4 and were allowed to watch the video/play the game as many times as they wanted for a total time of 20 min.

The video was developed based on a previously recorded Keynote presentation (version 6.6.2, Apple Inc.) with the addition of voice-over narration of the events. The video was edited to contain the same information as provided in the serious game, including the step-by-step care of a cardiac arrest victim. Its edited version lasted 3 min and 25 s.

The serious game was developed in a 3D learning environment simulating an urban public space where the player should identify a victim of cardiac arrest and perform CPR manoeuvres to help the victim (game demonstration available at https://www.youtube.com/watch?v=kB7amGKZM4k). The game was designed to be a CPR self-learning tool for both health professionals and the lay public. The game involved only one rescuer without access to a portable defibrillator. During the game, the player should identify the victim, reach a correct diagnosis of cardiac arrest, and initiate CPR as early as possible. The actions of the player were guided throughout the game by step-by-step instructions that should be followed to save the victim’s life and to score on the game. Whenever the manoeuvres were not performed correctly, the victim died and the game automatically restarted from the beginning.

Immediately after exposure to the self-learning methods, participants were reassessed individually for their theoretical knowledge on a written, 10-question, multiple-choice test (theoretical post-test) and for their practical performance by three examiners, blinded to group assignment, who independently rated the participants’ actions on a 10-min simulated scenario of cardiac arrest using a CPR training manikin (practical post-test).

Both the 10-item test for theoretical evaluation (S1 Questionnaire) and the 10-item checklist for practical evaluation (S2 Checklist), applied before and after the interventions, were adapted from the 2016 AHA Adult CPR and Automated External Defibrillator (AED) Skills Testing Checklist [25].

### Description of the simulated scenario

One unique simulated scenario was used for pre-exposure and post-exposure practical evaluation. In the simulation, students were required to care for a 50-year-old man possibly suffering from cardiac arrest. When the student entered the simulation room, a manikin (Little Anne model 120–01050; Laerdal Medical) was lying on the floor, simulating a person lying on the street, unconscious, not responding to stimulation and with no respiratory effort or pulse. The student was alone without access to medical equipment such as a defibrillator. We chose to reproduce such a scenario, with only one rescuer and no access to medical equipment, because this is the most common scenario in developing countries, such as Brazil, where there is no effective public policy focused on mass CPR training and AEDs are often not available in public spaces. Participants were expected to recognise the cardiac arrest, call the emergency services, and initiate chest compressions and ventilations as soon as possible for at least two cycles. A 10-min time limit was set for each simulation session.

### Data collection and outcomes

The three examiners involved in the rating of participants’ actions are duly certified general surgeons with more than 5 years’ experience in emergency care. The examiners rated the participants’ actions based on the sequence of steps on the 10-item checklist for practical evaluation (S2 Checklist). Points were given for actions performed correctly in sequence, and for the effectiveness and quality of chest compressions.

The manikin used in the simulation had a “clicker” feature that signalled the correct compression depth of 5 cm. For the purposes of this study, a chest compression was considered valid if the compression produced the audible feedback (“click”) indicating that the correct depth of 5 cm was achieved. The examiner gave 1 point for correct overall performance if compression produced an audible click in 50–75% of well-executed attempts, and 0 (zero) if there was an audible click in <50% of attempts. A video camera (Sony Cyber-shot, model DSC-HX5V, 2010) was used to record the simulated scenarios, and the recordings were later checked if there was any doubt about the actions of the participants.

The main outcome was the mean performance score of students on each (theoretical and practical) test for each study arm.

### Sample size

We planned a study of a continuous response variable from independent control and experimental subjects with 1 control per experimental subject. This was a pilot study, and there was no previous estimation of effect size regarding the compared interventions and the outcomes assessed. We then arbitrated that a difference between experimental and control means of 1 point in the performance score and a standard deviation (SD) of 1 unit would be clinically relevant effect size estimates. This resulted in an estimated sample size of 22 experimental subjects and 22 controls required to achieve a statistical power of 0.9 at a two-sided significance level (α) of 0.05.

### Statistical analysis

Continuous data were expressed as mean and SD. Categorical variables were expressed as counts and percentages. The primary endpoint was change in theoretical and practical baseline scores during simulated CPR. An analysis of covariance (ANCOVA) model was used to compare final scores between groups adjusting for baseline measurements. Differences between groups in time spent in self-directed learning were compared using Student’s *t* test. Additionally, all items comprising the score were evaluated as binary variables and compared between groups using Fisher’s exact test. We applied a Bonferroni correction to allow for multiple (22) comparisons. Therefore, a *p* value < 0.0022 (0.05/22) was considered significant. Data were analysed using SPSS, version 22.0.

## Results

A total of 45 participants were included in the study and randomly assigned to the video group (*n* = 22) or to the game group (*n* = 23). All participants completed the trial and were included in the data analysis. Most participants were women (62.2%). The baseline demographic characteristics and theoretical/practical scores were similar in the two groups (Table 1).

**Table 1:** Baseline characteristics of the participants, Porto Alegre, 2017.

Characteristics	Video group (*n* = 22)	Game group (*n* = 23)
Age, years, mean±SD	21.6±2.63	21.9±2.63
Female, *n* (%)	13 (59.1)	15 (65.2)
Theoretical pre-test score, mean±SD	4.91±1.93	4.65±2.19
Practical pre-test score, mean±SD	4.74±2.77	4.91±2.57

SD, standard deviation.

The mean performance scores of students on the theoretical and practical tests performed after exposure, for each study arm, are shown in Table 2. The video-based self-learning method was statistically superior to the serious game self-learning method in terms of knowledge acquisition. However, students remained interested in the game method for a longer time (Table 2).

**Table 2:** Outcome comparison between the video and game groups, Porto Alegre, 2017.

Outcomes	Video group (*n* = 22)	Game group (*n* = 23)	Adjusted difference (95%CI)	*p*
Theoretical post-test score^a^	7.56±0.21	6.51±0.21	1.05 (0.45–1.66)	0.001
Practical post-test score^a^	9.67±0.21	8.40±0.21	1.27 (0.67–1.87)	<0.001
Time, min^b^	7.41±0.43	18.57±0.66	− 14.05 (−12.76 to −9.55)	<0.001

Data are presented as mean ± standard error.

^a^ Analysis of covariance (ANCOVA) model adjusted for baseline measurements.

^b^ Student’s *t* test.

When students’ theoretical knowledge was evaluated in relation to each one of the 10 questions comprising the theoretical post-test, the video group performed better in almost all questions. Only in question #2 (‘If you find an unconscious person, what is the first thing to do?’), however, there was a statistically significant difference between the two groups (*p* = 0.002) (Table 3).

**Table 3:** Comparison of correct answers given to individual questions between the video and game groups—theoretical post-test, Porto Alegre, 2017.

Questions	Video group (*n* = 22)	Game group (*n* = 23)	*p*
1. From the options below, which is the best indicator that a person is having a cardiac arrest?	95.5%	91.3%	>0.999
2. If you find an unconscious person, what is the first thing to do?	81.8%	34.8%	**0.002**
3. If the person loses consciousness and does not respond to any stimuli, what is the first thing to do?	68.2%	56.5%	0.542
4. When you call emergency services, what kind of information should you provide?	68.2%	73.9%	0.749
5. When performing CPR, what kind of protocol should you follow?	100%	91.3%	0.489
6. While performing mouth-to-mouth ventilations (breaths), which of these signs ensures that the procedure was performed correctly?	77.3%	60.9%	0.337
7. While giving chest compressions, which of these signs ensures that the procedure was performed correctly?	95.5%	78.3%	0.187
8. Considering that A = Airway, B = Breathing, and C = Compression, what is the correct sequence of steps to perform CPR manoeuvres currently?	59.1%	52.2%	0.767
9. What is the best hand position for chest compression?	100%	100%	—
10. If the person does not respond to any stimuli, what is the worst thing to do?	9.1%	13.0%	>0.999

In the practical post-test, students in the video group also performed better in most of the 10 items comprising the checklist used by the examiners. However, only item #1 (‘Did not check responsiveness’) showed a statistically significant difference between the two groups (*p* < 0.001) (Table 4).

**Table 4:** Comparison of checklist items between the video and game groups—practical post-test, Porto Alegre, 2017.

Items	Video group (*n* = 22)	Game group (*n* = 23)	*p*
1. Did not check responsiveness	0	56.5%	**<0.001**
2. Did not shout for help—call emergency services	0	13.0%	0.233
3. Did not check breathing and pulse	0	4.3%	>0.999
4. Did not place the hands correctly on the patient’s chest	9.1%	4.3%	0.608
5. Did not start the first compression cycle correctly	0	8.7%	0.489
6. Did not compress the chest correctly	0	13.0%	0.233
7. Did not allow the chest to recoil completely before the next compression	0	17.4%	0.109
8. Did not perform mouth-to-mouth ventilation correctly	0	17.4%	0.109
9. Did not perform the second compression cycle	9.1%	4.3%	0.608
10. Did not perform the second mouth-to-mouth ventilation cycle correctly	18.2%	17.4%	>0.999

## Discussion

Although serious games have been advocated as more effective than other self-learning methods [26], our research suggests quite the opposite. We expected the theoretical and practical CPR performance to be higher in the game group, given students’ clear preference for using games as observed in this and previous studies [19, 20]. Instead, average CPR performance in the game group was lower than in the video group after a short period of exposure.

Students seem to remain interested in the serious game longer than in the video-based course. Serious games are known to be motivating and strongly engaging for students, stimulating them to study longer; however, their use has not resulted in improved patient safety knowledge and awareness compared to video-recorded lectures [26, 27]. Consistent with these findings, inexperienced medical students remained using the serious game used in the present study for a longer time, suggesting that this method is more engaging than the video-based course, but it did not result in improved performance. This raises an important question as to whether resources should be allocated for the development of self-learning tools that are more engaging for students, such as serious games, or that may be more useful in helping students retain skills, such as video-based on-line courses.

Providing high-quality chest compression is the most important action in CPR procedures to improve patient’s outcome [28, 29]. However, it requires hands-on sessions for proper skill acquisition, preferably with the use of a feedback system [30–32]. Cortegiani et al. [32], evaluating secondary students training on chest compressions with an instructor and a real-time electronic feedback system (Laerdal QCPR), suggested that feedback from software may improve technical acquisition on the ability to perform chest compressions with adequate recoil compared with training with standard instructor-based feedback only. The Little Annemodel 120–01050 used in the present study has been discontinued by the manufacturer and replaced by Little Anne QCPR. It is possible to upgrade this manikin model to include QCPR feedback technology, which we believe would provide a more reliable assessment of students’ performance. Nevertheless, although we did not use software-based metrics to evaluate the effectiveness of compression in achieving the correct depth of 5 cm, the quality of chest compressions improved in both groups after exposure, as determined by an audible click, despite the 13% rate of incorrectly performed compressions still observed in the game group.

Overall, students in the video group obtained higher scores in all items of the sequence of steps required for the management of cardiac arrest. Both methods included all stages from recognition to management of cardiac arrest. The video initially provided an overview of the clinical diagnosis and then presented the steps of management, ending with a summary of actions. In the serious game, however, students needed to recognise the cardiac arrest and perform actions without an initial overview of the procedure. Instead, at each stage, players were interrupted by instructions, which could be skipped, allowing them to continue playing in a trial-and-error fashion. Therefore, the lower performance of students in the game group may be attributed to the fact that players gave little attention to the instructions that they had received, thereby playing the game in a more enjoyable way, making attempts without prior planning. We believe that providing initial instructions on the adequate sequence of actions to perform and using a brief instructional video as a preparation for the self-training game as well as before decision-making situations may increase knowledge acquisition, although it may conversely decrease the student’s interest in playing the game due to breaking game continuity.

Developing serious games is more expensive and time-consuming than using video-based methods. In the present study, the sequence of CPR manoeuvres to be performed when managing cardiac arrest was better acknowledged by students in the video group, although students in the game group were more interested in the method. Therefore, a next step for future trials would be the comparison of self-learning models that combine the advantages of the two methods, which may increase students’ performance and interest in the CPR content compared to the use of video or serious game alone.

This study has several limitations. First, the comparison of the two self-learning methods would have benefited from the inclusion of a control group using a traditional self-learning method, such as textbooks, in order to confirm the effectiveness of digital methods over traditional methods [33]. Second, data on later assessment (30 days) after exposure were not presented. Third, the specific serious game tested in the present study has not been previously validated, which limits the generalisation of our findings to settings with different serious games or target population. That is, despite using the same study design, the use of different videos and games may produce conflicting results. However, readers can gain a better understanding of the features and functions of the video and serious game used in our study by watching a demonstration video available on the internet (https://www.youtube.com/watch?v=kB7amGKZM4k). Fourth, this is a single-centre study, which also limits the generalisation of the results. Fifth, because this is a simulation-based study, the performance results may not be generalised to real-life situations. Finally, we did not use special software to evaluate the quality of chest compressions. Instead, the effectiveness and quality of chest compressions were evaluated by direct observation by the examiners. Although examiners were allowed to review the recorded simulation sessions whenever there was any doubt about the actions of the participants, this rating system is known to be less accurate than chest compression software systems [24, 32]. Also, we used both video-based and 3D games as a self-learning method rather than an adjunct to an instructor-guided, manikin-based simulation training, which currently represents the most effective and widely adopted CPR training method. In this respect, the trial design adopted by Drummond et al. [24], in which serious game was compared to a PowerPoint lecture as a pre-training adjunct was more adherent to current CPR training guidelines. The authors also noticed that the most difficult skill to acquire is high-quality chest compression, even with the use of an instructor-based training [24].

## Conclusions

The self-training modality using a serious game, after a short period of exposure, resulted in inferior students’ performance in both theoretical and practical CPR tests compared to the video-based self-training modality. However, students seem to be more interested in using games rather than videos as a form of self-training.

Although in both groups students improved their CPR performance scores after exposure, we cannot ensure that, in real-life situations, such performance will be as effective as that of students who have received face-to-face instructor-led training or had access to real-time CPR feedback devices. We believe that these two self-training modalities are useful in helping learners and may be used as an introductory method for CPR training, since they are easily accessible from a smartphone and can be integrated into face-to-face training courses for health professionals and even for lay rescuers.

## Supporting information

S1 Checklist Simulation-Based Research Extensions to the CONSORT Statement. (DOCX)

S2 Checklist 10-item checklist for evaluation of practical performance (Portuguese and English versions). (DOCX)

S1 Questionnaire 10-item, multiple-choice questionnaire for evaluation of theoretical knowledge (Portuguese and English versions). (DOCX)

S1 Document Commission for Evaluation of Scientific Productivity, Pontifícia Universidade Católica do Rio Grande do Sul (PUCRS). (PDF)
