# Supplementary material for: Comparative evaluation of video-based on-line course versus serious game for training medical students in cardiopulmonary resuscitation: A randomised trial
Source: PLoS One. 2019 Apr 8;14(4):e0214722. doi: 10.1371/journal.pone.0214722 (PMC6453387; doi:10.1371/journal.pone.0214722)
Supplement: S1 Checklist — (DOCX) [file pone.0214722.s001.docx]

Simulation-Based Research Extensions to the CONSORT Statement

| **Item** | **Item number** | **CONSORT description (Randomised Controlled Trials)** | **Extension for SBR** | **Reported on page No.** |
| --- | --- | --- | --- | --- |
| Title and abstract | 1 | a. Identification as a randomised trial in the title  b. Structured summary of trial design, methods, results, and conclusions | In abstract or key terms, the MESH or searchable keyword term must have the word “simulation” or “simulated.” | 1  2 |
| Introduction | | | |  |
| Background | 2 | a. Scientific background and explanation of rationale  b. Specific objectives or hypotheses | Clarify whether simulation is subject of research or investigational method for research. | 3,4  4 |
| Methods | | | |  |
| Trial design | 3 | a. Description of trial design (such as parallel, factorial) including allocation ratio  b. Important changes to methods after trial commencement (such as eligibility criteria), with reasons |  | 4  N/A |
| Participants | 4 | a. Eligibility criteria for participants  b. Settings and locations where the data were collected |  | 4,5  4 |
| Interventions | 5 | The interventions for each group with sufficient details to allow for replication, including how and when they were actually administered. | Describe the theoretical and/or conceptual rationale for the design of each intervention.  Clearly describe all simulation-specific exposures, potential confounders, and effect modifiers. | 5-7  6 |
| Outcomes | 6 | a. Completely defined prespecified primary and secondary outcome measures, including how and when they were assessed  b. Any changes to trial outcomes after the trial commenced, with reasons | In describing the details of methods of assessment, include (when applicable) the setting, instrument, simulator type, timing in relation to the intervention, along with any methods used to enhance the quality of measurements.  Provide evidence to support the validity and reliability of assessment tools in this context (if available). | 7,8  N/A |
| Sample size/study size | 7 | a. How sample size was determined  b. When applicable, explanation of any interim analyses and stopping guidelines |  | 8  N/A |
| Randomization: sequence generation | 8 | a. Method used to generate the random allocation sequence  b. Type of randomization and details of any restriction (such as blocking and block size) |  | 5  5 |
| Randomization: allocation concealment mechanism | 9 | Mechanism used to implement the random allocation sequence (such as sequentially numbered containers), describing any steps taken to conceal the sequence until interventions were assigned |  | 5 |
| Randomization: implementation | 10 | Who generated the random allocation sequence, who enrolled participants, and who assigned participants to interventions |  | 5 |
| Blinding (masking) | 11 | a. If done, who was blinded after assignments to interventions (e.g., participants, care providers, those assessing outcomes) and how  b. If relevant, description of the similarity of interventions | Describe strategies to decrease risk of bias, when blinding is not possible. | 6  6 |
| Statistical methods | 12 | a. Statistical methods used to compare groups for primary and secondary outcomes  b. Methods for additional analyses, such as subgroup analyses and adjusted analyses | Clearly indicate the unit of analysis (e.g., individual, team, system), identify repeated measures on subjects, and describe how these issues were addressed. | 8,9  8,9 |
| Results | | | |  |
| Participant flow (a diagram is strongly recommended) | 13 | a. For each group, the numbers of participants who were randomly assigned, received intended treatment, and were analysed for the primary outcome  b. For each group, losses and exclusions after randomization, together with reasons |  | 9  N/A |
| Recruitment | 14 | a. Dates defining the periods of recruitment and follow-up  b. Why the trial ended or was stopped |  | 4  N/A |
| Baseline data | 15 | A table showing baseline demographic and clinical characteristics of each group | In describing characteristics of study participants, include their previous experience with simulation and other relevant features as related to the intervention(s). | 9 |
| Numbers analysed | 16 | For each group, number of participants (denominator) included in each analysis and whether analysis was by original assigned groups |  | 9 |
| Outcomes and estimation | 17 | a. For each primary and secondary outcome, results for each group, and the estimated effect size and its precision (such as 95 % confidence interval)  b. For binary outcomes, presentation of both absolute and relative effect sizes is recommended | For assessments involving >1 rater, interrater reliability should be reported. | 10  N/A |
| Ancillary analyses | 18 | Results of any other analyses performed, including subgroup analyses and adjusted analyses, distinguishing prespecified from exploratory |  | 11-14 |
| Adverse events | 19 | All important harms or unintended effects in each group (for specific guidance, see CONSORT for harms) |  | N/A |
| Discussion | | | |  |
| Limitations | 20 | Trial limitations, addressing sources of potential bias, imprecision, and, if relevant, multiplicity of analyses | Specifically discuss the limitations of SBR. | 17,18 |
| Generalizability | 21 | Generalizability (external validity, applicability) of the trial findings | Describe generalizability of simulation-based outcomes to patient-based outcomes (if applicable). | 15,18 |
| Interpretation | 22 | Interpretation consistent with results, balancing benefits and harms, and considering other relevant evidence |  | 16,17 |
| Other information | | | |  |
| Registration | 23 | Registration number and name of trial registry |  | 4 |
| Protocol | 24 | Where the full trial protocol can be accessed, if available |  | N/A |
| Funding | 25 | Sources of funding and other support (such as supply of drugs), role of funders | List simulator brand and if conflict of interest for intellectual property exists. | 25 |

*MESH* Medical Subject Headings
