# Supplementary material for: Comparative evaluation of video-based on-line course versus serious game for training medical students in cardiopulmonary resuscitation: A randomised trial
Source: PLoS One. 2019 Apr 8;14(4):e0214722. doi: 10.1371/journal.pone.0214722 (PMC6453387; doi:10.1371/journal.pone.0214722)
Supplement: S2 Checklist — (DOCX) [file pone.0214722.s002.docx]

**S3 Checklist.** 10-item checklist for evaluation of practical performance (Portuguese and English versions).

Pré-teste / Pós-teste

|  | **Lista de verificação para atendimento de paciente adulto. Protocolo de atendimento em RCP para um socorrista.** | Correto  Ciclo 01 | Correto  Ciclo 02 |
| --- | --- | --- | --- |
| **1** | **Checar por resposta**  (balançar os ombros, gritar em voz alta) |  |  |
| **2** | **Gritar por ajuda - Chamar 192** |  |  |
| **3** | **Checar respiração e pulso**  (por pelo menos 5 segundos e não mais que 10 segundos) |  |  |
|  | **Iniciar manobras de RCP** |  |  |
| **4** | **Colocar mãos corretamente no tórax**  (2/3 inferiores do tórax) |  |  |
| **5** | **Iniciar primeiro ciclo de compressões**  *Aceitável < 18 segundos para 30 compressões* |  |  |
| **6** | **Compressão adequada**  *Deformar o tórax em 5 cm para pelo menos 23/30 compressões* |  |  |
| **7** | **Permitir completo retorno do tórax antes de uma nova compressão**  *Em pelo menos 23/30 compressões* |  |  |
| **8** | **Minimizar interrupções: Fazer 2 respirações em menos de 10 segundos**  *Abrir a via aérea e fazer 2 respirações (1 segundo cada)* |  |  |
| **9** | **Fazer segundo ciclo de compressões com a correta posição das mãos**  *Aceitável >23 de 30 compressões* |  |  |
| **10** | **Fazer 2 respirações (1 segundo cada)** |  |  |

Adaptado de American Heart Association (AHA) Adult CPR and AED Skills Testing Checklist 2016.

Pre-test / Post-test

|  | **Checklist for adult patient care. CPR care protocol for rescuers.** | Correct  Cycle 01 | Correct  Cycle 02 |
| --- | --- | --- | --- |
| **1** | **Checks responsiveness**  (shakes shoulders, shouts out loud) |  |  |
| **2** | **Shouts for help - Calls 911** |  |  |
| **3** | **Checks breathing and pulse**  (for at least 5 seconds and no longer than 10 seconds) |  |  |
|  | **Begins CPR manoeuvres** |  |  |
| **4** | **Places the hands correctly on the chest**  (2/3 lower chest) |  |  |
| **5** | **Begins first cycle of compressions**  *Acceptable <18 seconds for 30 compressions* |  |  |
| **6** | **Adequate compression**  *Chest is depressed to 5 cm in at least 23/30 compressions* |  |  |
| **7** | **Allows complete chest recoil before a new compression**  *In at least 23/30 compressions* |  |  |
| **8** | **Minimizing interruptions: Gives 2 breaths in less than 10 seconds**  *Opens airway and gives 2 breaths (1 second each)* |  |  |
| **9** | **Performs second cycle of compressions with correct hand placement**  *Acceptable >23 out of 30 compressions* |  |  |
| **10** | **Gives 2 breaths (1 second each)** |  |  |

Adapted from the 2016 American Heart Association (AHA) Adult CPR and AED Skills Testing Checklist.
