# Supplementary material for: Comparative evaluation of video-based on-line course versus serious game for training medical students in cardiopulmonary resuscitation: A randomised trial
Source: PLoS One. 2019 Apr 8;14(4):e0214722. doi: 10.1371/journal.pone.0214722 (PMC6453387; doi:10.1371/journal.pone.0214722)
Supplement: S1 Questionnaire — (DOCX) [file pone.0214722.s003.docx]

**S2 Questionnaire.** 10-item, multiple-choice questionnaire for evaluation of theoretical knowledge (Portuguese and English versions).

Pré-teste / Pós-teste

| TESTE DE CONHECIMENTO - Ressuscitação cardiopulmonar (RCP) | |
| --- | --- |
| **1. Das opções abaixo qual o melhor indicador de que uma pessoa está sofrendo uma parada cardíaca?**  a. Intensa dor no peito  b. Intensa dificuldade respiratória  c. Pessoa não consegue se manter em pé  d. Pessoa não responde ao chamado e não está respirando | **6. Quando você faz uma respiração boca-a-boca(sopro) qual sinal lhe garante que foi feita corretamente?**  a. O ar flui facilmente  b. Você ouve o som da respiração a cada sopro  c. O tórax da pessoa se eleva a cada sopro  d. A pessoa fica azulada |
| **2. Encontrando uma pessoa desacordada qual a primeira coisa a ser feita?**  a. Ligar para 192  b. Chacoalhar os ombros dela e perguntar em voz alta se a pessoa está bem  c. Iniciar choque com desfibrilador  d. Fazer dois ciclos de ressuscitação cardiopulmonar e ver se a pessoa responde | **7. Quando você faz uma massagem cardíaca qual sinal lhe garante que foi feita corretamente?**  a. O tórax deforma em pelo menos 5 cm  b. Consegue ouvir as costelas quebrando  c. Os vasos do pescoço ficam saltados  d. A pessoa tem espasmos |
| **3. Quando a pessoa não responde e não acorda a estímulo, qual a primeira coisa a ser feita?**  a. Não fazer nada até a chegada de um desfibrilador  b. Verificar se a pessoa está respirando e com pulso e iniciar as manobras de ressuscitação  c. Ligar para 192 e pedir ajuda  d. Colocar uma compressa de gelo na testa da pessoa para melhorar sua resposta | **8. Sabendo que A = Abertura das vias aéreas , B = Respiração e C = Compressão, qual a sequência correta para realizar as manobras de ressuscitação cardiopulmonar atualmente?**  a. A - B - C  b. C - A - B  c. B - A - C  d. C - B - A |
| **4. Quando você ligar para 192 que tipo de informação você deve fornecer?**  a. Seu nome e localização  b. Tipo de emergência  c. O número de telefone que você está ligando  d. a, b e c | **9. Qual o melhor posicionamento das mãos para massagem cardíaca?**  a. Dedos entrelaçados logo abaixo do pescoço  b. Mãos espalmadas linha dos mamilos  c. Dedos entrelaçados linha dos mamilos  d. Mãos espalmadas logo abaixo do pescoço |
| **5. Quando estiver realizando uma ressuscitação cardiopulmonar que tipo de protocolo você deve seguir?**  a. 15 compressões para 2 respirações boca-boca, repetir  b. 05 compressões para 1 respirações boca-boca, repetir  c. 15 compressões para 5 respirações boca-boca, repetir  d. 30 compressões para 2 respirações boca-boca, repetir | **10. Quando uma pessoa não responde e não acorda a estímulo, qual a pior coisa que você pode fazer?**  a. Tentar fazer uma ressuscitação cardiopulmonar sem ter certeza como fazer corretamente  b. Usar um desfibrilador sem ter certeza como fazer corretamente  c. Realizar a massagem cardíaca sem fazer a respiração por nojo ou medo de fazer respiração boca-a-boca  d. Pedir ajuda e aguardar por alguém mais qualificado e enquanto isso proteger a pessoa |

Adaptado de American Heart Association (AHA) Adult CPR and AED Skills Testing Critical Skills Descriptors 2016.

Pre-test / Post-test

| KNOWLEDGE TEST - Cardiopulmonary resuscitation (CPR) | |
| --- | --- |
| **1. From the options below, which is the best indicator that a person is having a cardiac arrest?**  a. Severe chest pain  b. Severe respiratory distress  c. The person cannot stand upright  d. The person is unresponsive with absent breathing | **6. While performing mouth-to-mouth ventilations (breaths), which of these signs ensures that the procedure was performed correctly?**  a. The air flows easily  b. You can hear the sound of breathing with each breath delivered  c. Each breath delivered causes chest rise  d. The skin becomes bluish |
| **2. If you find an unconscious person, what is the first thing to do?**  a. Call 911  b. Shake the person’s shoulders and ask out loud if the person is all right  c. Begin defibrillation  d. Perform two cycles of CPR and check responsiveness | **7. While giving chest compressions, which of these signs ensures that the procedure was performed correctly?**  a. The chest is depressed at least 5 cm  b. You can hear the ribs breaking  c. The neck vessels are prominent  d. The person has spasms |
| **3. If the person loses consciousness and does not respond to any stimuli, what is the first thing to do?**  a. Do nothing until a defibrillator is available  b. Check breathing and pulse and begin CPR manoeuvres  c. Call 911 and ask for help  d. Apply an ice pack to the person’s forehead to improve responsiveness | **8. Considering that A = Airway, B = Breathing, and C = Compression, what is the correct sequence of steps to perform CPR manoeuvres currently?**  a. A - B - C  b. C - A - B  c. B - A - C  d. C - B - A |
| **4. When you call 911, what kind of information should you provide?**  a. Your name and location  b. Type of emergency  c. The phone number you are calling from  d. a, b, and c | **9. What is the best hand position for chest compression?**  a. Fingers interlaced just below the neck  b. Palms of the hands on the nipple line  c. Fingers interlaced on the nipple line  d. Palms of the hands just below the neck |
| **5. When performing CPR, what kind of protocol should you follow?**  a. 15 compressions to 2 breaths, repeat  b. 5 compressions to 1 breath, repeat  c. 15 compressions to 5 breaths, repeat  d. 30 compressions to 2 breaths, repeat | **10. If the person does not respond to any stimuli, what is the worst thing to do?**  a. Attempting CPR while being unsure how to perform it correctly  b. Using a defibrillator while being unsure how to use it correctly  c. Giving chest compressions not followed by breaths because of disgust or fear of mouth-to-mouth contact  d. Asking for help and waiting for qualified rescuers while protecting the person |

Adapted from the 2016 American Heart Association (AHA) Adult CPR and AED Skills Testing Critical Skills Descriptors.
