# Supplementary material for: Comparative evaluation of video-based on-line course versus serious game for training medical students in cardiopulmonary resuscitation: A randomised trial
Source: PLoS One. 2019 Apr 8;14(4):e0214722. doi: 10.1371/journal.pone.0214722 (PMC6453387; doi:10.1371/journal.pone.0214722)
Supplement: S1 Document — (PDF) [file pone.0214722.s004.pdf]

S I P E S Q

Sistema de Pesquisas da PUCRS

Porto Alegre, 28 de julho de 2014.

Documento unificado referente ao projeto de pesquisa:

5864 - Avaliação comparativa de performace entre e-learning e jogos de computador em manobras de parada cardiorespiratória

Este documento unificado é composto por:

Arquivos	Página
- Carta de Aprovacao da Comissao Cientifica	2
- Projeto pesquisa	3
- Orçamento	8

Código SIPESQ: 5864 Porto Alegre, 28 de julho de 2014.

Prezado(a) Pesquisador(a),

A Comissão Científica da FACULDADE DE MEDICINA da PUCRS apreciou e aprovou o Projeto de Pesquisa "Avaliação comparativa de performace entre e-learning e jogos de computador em manobras de parada cardiorespiratória" coordenado por VINICIUS DUVAL DA SILVA. Caso este projeto necessite apreciação do Comitê de Ética em Pesquisa (CEP) e/ou da Comissão de Ética no Uso de Animais (CEUA), toda a documentação anexa deve ser idêntica à documentação enviada ao CEP/CEUA, juntamente com o Documento Unificado gerado pelo SIPESQ.

Atenciosamente,

Comissão Científica da FACULDADE DE MEDICINA

PONTIFÍCIA UNIVERSIDADE CATÓLICA DO RIO GRANDE DO SUL

CURSO DE PÓS-GRADUAÇÃO EM MEDICINA E CIÊNCIAS DA SAÚDE

FAMED/PUCRS

Projeto de Pesquisa

ÁREA DE CONCENTRAÇÃO

CLINICA MÉDICA

LINHA DE PESQUISA

FORMAÇÃO E DESENVOLVIMENTO DOCENTE NA SAÚDE

Avaliação comparativa de performance entre e-learning e jogos de computador em manobras de parada cardiorrespiratória

Pesquisadores Principais:

David Ponciano de Sena

Vinicius Duval da Silva

Porto Alegre, maio de 2014.

Sistema de Pesquisas da PUCRS
SIPESQ

1. Introdução

Nos últimos anos tem-se observado um revolução na maneira com que as pessoas se relacionam, produzem e consomem conteúdo. O aparecimento de ferramentas gratuitas como facebook, youtube, slideshare, twitter e outras demais plataformas de troca de informações são amplamente utilizadas para interação interpessoal, e já de uma forma incipiente para trabalho, contudo, ainda pouco exploradas para gerar conteúdo científico e de educação.

Sabe-se ainda que a cada ano, milhares de pessoas são acometidas por parada cardiorrespiratória e que um indivíduo adequadamente treinado em manobras de ressuscitação cardiopulmonar poderia aumentar significativamente a sobrevida desses pacientes. Dessa forma, qualquer iniciativa que venha a oferecer um treinamento de fácil entendimento e comprovada eficácia provavelmente teria um impacto positivo no atendimento de uma eventual vítima.

Nesse novo contexto de interação com realidade virtual, simulações computadorizadas em programas de ensino assistidos por computador ou CAL (*Computer Assisted Learning*) para aprendizagem, como o e-learning (ensino a distância pela internet) e a gamificação (utilização de jogos para ensino) vem despontando como alternativas eficientes ao ensino não presencial de conteúdo educativo científico.

Nesses modelos de ensino, os alunos podem aprender as informações por conta própria, independente da presença de um instrutor para obter boa performance principalmente em situações de procedimentos básicos. Nessas situações, já se verificou que o desempenho de alunos que utilizam instrução baseada em computadores obtém o mesmo resultado de alunos orientados por instrutores e superior aos baseados apenas em livro texto, sabendo-se contudo, que aqueles que recebem *feedback* no processo de aprendizagem ou tutoria ainda apresentam um melhor resultado final.

2. Objetivos

2.1 Geral. Criação de duas plataformas utilizando conceitos de e-learning e criação de jogos interativos (gamificação) para condutas em uma parada cardiorrespiratória.

2.2 Específico. Desenvolvimento, implementação e avaliação e comparação da eficácia das duas plataformas simples e amigáveis, que agreguem conceitos de e-learning e gamificação respectivamente para a exploração do conteúdo de condutas em uma parada cardiorrespiratória.

SIPESQ Sistema de Pesquisas da PUCRS

3. Métodos

Alunos do curso técnico de enfermagem do Hospital Moinhos de Ventos, serão convidados a participar do estudo de forma voluntária preenchendo um consentimento informado prévio sobre o caráter da pesquisa e garantindo a confidencialidade dos dados e resultados individuais. Todos os participantes vão ser submetidos a um pré-teste baseado nas diretrizes da AHA(American Heart Association) referente ao ano de 2010 , consistindo em 30 perguntas com 4 opções de escolha sendo apenas uma correta, acerca de seus conhecimentos prévios sobre conduta em parada cardiorrespiratória. Após o pré-teste os alunos serão subdivididos e alocados em uma relação de 1:1:1 em três grupos de forma randomizada contendo um total de 40 alunos para cada grupo.

O grupo um (controle - G1) será submetido a uma aula expositiva sobre conduta em parada cardiorrespiratória, o grupo dois (e-learning - G2) submetido a treinamento individual assistido por computador em formato multimídia (elearning) e o grupo três (jogo de computador - G3) a treinamento individual assistido por computador em formato de jogo de simulação com interação direta na tela. Cada grupo será submetido a período médio de exposição de 30 minutos, sendo todo o conteúdo baseado nas diretrizes do AHA 2010.

Após a exposição, cada participante será submetido a um pós-teste e a uma avaliação prática individual de conduta perante uma PCR em modelo anatômico que será filmado e posteriormente avaliado por 03 avaliadores cegados utilizando uma lista de checagem de 14 itens sequenciais já validado previamente por estudos anteriores. O alunos participantes dos grupos que utilizarem instrução por e-learning e jogo de computador serão convidados a preencher uma avaliação de satisfação sobre os métodos a que foram submetidos.

Passado 06 meses do treinamento os participantes serão reavaliados de forma teórica e prática afim de determinar a retenção do conhecimento.

A análise para dados ordinários será realizada utilizando o software SPSS versão 10.0, aplicando o teste de Mann-Whitney para dados ordinários e o teste Chi-quadrado para dados categóricos, admitindo-se $p<0,05$ como valor com significância estatística. O tamanho de amostra foi determinado levando em conta a necessidade de detectar diferença de 5% com 90% de poder de significância de 0,05.

A pesquisa será devidamente registrada na base de dados nacional de pesquisas

SIPESQ Sistema de Pesquisas da PUCRS

em andamento pela Plataforma Brasil e passará pela avaliação comitê de ética em pesquisa do Hospital São Lucas da PUCRS e Hospital Moinhos de Ventos.

4. Orçamento

Todo o gasto com desenvolvimento, aplicação e implementação será financiado pelos pesquisadores e parceiros, não acarretando custos adicionais à PUCRS.

5. Cronograma

Período/ Atividade	08/13 – 10/13	11/13 - 01/14	02/14 – 04/14	05/14 – 07/14	08/14 – 10/14	11/14 – 01/15	02/15 – 04/15	05/15 – 07/15	08/15 – 10/15	11/15 – 01/16	02/16 – 04/16	05/16 – 07/16
Revisão da literatura	X	X	X	X	X	X	X	X	X	X	X	X
Envio/ Aprovação CEP			X	X	X	X						
Coleta de dados					X	X	X	X	X	X		
Estudo do material					X	X	X	X	X	X		
Análise dos resultados					X						X	
Redação para publicação						X						X

5. Referências Bibliográficas

1. BERG, R. A. et al. Part 5: adult basic life support: 2010 American Heart Association Guidelines for Cardiopulmonary Resuscitation and Emergency Cardiovascular Care. **Circulation**, v. 122, n. 18 Suppl 3, p. S685–705, 2 nov. 2010.

2. BRENNAN, R. T. et al. A reliable and valid method for evaluating cardiopulmonary resuscitation training outcomes. **Resuscitation**, v. 32, n. 2, p. 85–93, set. 1996.

SIPESQ Sistema de Pesquisas da PUCRS

3. CHOA, M. et al. The effectiveness of cardiopulmonary resuscitation instruction: animation versus dispatcher through a cellular phone. **Resuscitation**, v. 77, n. 1, p. 87–94, abr. 2008.

4. LEE, J. S. et al. The effect of a cellular-phone video demonstration to improve the quality of dispatcher-assisted chest compression-only cardiopulmonary resuscitation as compared with audio coaching. **Resuscitation**, v. 82, n. 1, p. 64–68, jan. 2011.

5. PAAL, P. et al. Effects of training time and feedback on ventilation skills in lay rescuers. **Emergency medicine journal: EMJ**, v. 27, n. 4, p. 313–316, abr. 2010.

6. YANG, C.-W. et al. Impact of adding video communication to dispatch instructions on the quality of rescue breathing in simulated cardiac arrests--a randomized controlled study. **Resuscitation**, v. 78, n. 3, p. 327–332, set. 2008.

7. YANG, C.-W. et al. Interactive video instruction improves the quality of dispatcher-assisted chest compression-only cardiopulmonary resuscitation in simulated cardiac arrests. **Critical care medicine**, v. 37, n. 2, p. 490–495, fev. 2009.

8. DE SENA, D. P. et al. Computer-assisted teaching of skin flap surgery: validation of a mobile platform software for medical students. **PloS one**, v. 8, n. 7, p. e65833, 2013.

SIPESQ
Sistema de Pesquisas da PUCRS

PUCRS

PROPESQ **CENTRO DE PESQUISA CLÍNICA**

HOSPITAL SÃO LUCAS DA PUCRS

** campos a serem preenchidos pelo CPC

	**número	**mês	**semestre	**ano
**ORÇAMENTO DO PROJETO**	10160	5	1	2014

TÍTULO DA PESQUISA: Avaliação comparativa de performance entre e-learning e jogos de computador em manobras de parada cardiorespitatória.

PESQUISADOR RESPONSÁVEL: Vinícius Duval da Silva

ITENS A SEREM FINANCIADOS	QUANTIDADE			VALOR	VALOR POR	VALOR	*Fonte
ESPECIFICAÇÕES	nº de Pacientes<br>nº de animais	nº de exames<br>nº de mat ou equip	total	UNITÁRIO R$	PACIENTE R$	TOTAL R$	Viabilizadora
	quantidades	quantidades					
**Exames/Procedimentos :**						0,00	
			0	0,00		0,00	
			0	0,00		0,00	
			0	0,00		0,00	
			0	0,00		0,00	
**Serviços:**			0	0,00		0,00	
módulo e-learning			0	0,00		0,00	
design gráfico	1	1	1	1000,00		1.000,00	6
animações multimídia	1	1	1	3000,00		3.000,00	6
gravação da narrativa em estúdio	1	1	1	1000,00		1.000,00	6
			0	0,00		0,00	
			0	0,00		0,00	
módulo jogo			0	0,00		0,00	
design gráfico	1	1	1	1000,00		1.000,00	6
desenvolvimento do personagem	1	1	1	1000,00		1.000,00	6
programação	1	1	1	3000,00		3.000,00	6
gravação da narrativa em estúdio	1	1	1	1000,00		1.000,00	6
			0	0,00		0,00	
			0	0,00		0,00	
			0	0,00		0,00	
			0	0,00		0,00	
			0	0,00		0,00	
**Materiais :**			0	0,00		0,00	
			0	0,00		0,00	
**Equipamentos:**			0	0,00		0,00	
			0	0,00		0,00	
			0	0,00		0,00	
			0	0,00		0,00	
			0	0,00		0,00	
			0	0,00		0,00	
			0	0,00		0,00	
**TOTAL**				**11.000,00**		11.000,00	

OBSERVAÇÕES:
anexar comprovante de cronograma de execução; anexar comprovante de Agência de Fomento (quando for o caso)

Prof. Dr. Vinícius Duval da Silva
Coordenador do Laboratório de Anatomia Patológica e Citopatologia
Hospital São Lucas da PUCRS

Dr. Domingos Otavio Lorenzoni D'Avila
Coordenador
Centro de Pesquisa Clínica
HSL-PUCRS

Dr. Álvaro de Castro A. Maciel
CREMERS 17388
CPF: 284.446.471-87

Ass. Pesquisador Responsável (assinatura e carimbo)	Ass. Coordenação do CPC (assinatura e carimbo)	Ass. Chefia do Serviço (assinatura e carimbo)	Ass. Direção HSL (assinatura e carimbo, qdo necessário)

*** Fontes viabilizadoras:**

1. HSL (Rotina da assistência)	3. Patrocinador	5. Serviço	7. Estudo Retrospectivo
2. HSL (Extra-rotina da assistência)	4. Agência de Fomento	6. Pesquisador	

DATA DE ELABORAÇÃO: 25/5/2014 — data de impressão: 26/5/2014

Prof. R[illegible] Henrique Duarte Martins
Coord. Adj. Centro de Pesquisas Clínicas
CPC-HSL-PUCRS

10160-01-2014
